# Fetal Muse-based therapy prevents lethal radio-induced gastrointestinal syndrome by intestinal regeneration

**DOI:** 10.1186/s13287-023-03425-1

**Published:** 2023-08-11

**Authors:** Honorine Dushime, Stéphanie G. Moreno, Christine Linard, Annie Adrait, Yohann Couté, Juliette Peltzer, Sébastien Messiaen, Claire Torres, Lydia Bensemmane, Daniel Lewandowski, Paul-Henri Romeo, Vanessa Petit, Nathalie Gault

**Affiliations:** 1 Université Paris Cité, Inserm, CEA, Stabilité Génétique Cellules Souches et Radiations, Laboratoire Réparation et Transcription dans les cellules Souches (LRTS), Institut de Radiobiologie Cellulaire et Moléculaire (iRCM), Institut de Biologie François Jacob (IBFJ), CEA, 92260 Fontenay-aux-Roses, France; 2 Université Paris-Saclay, Inserm, CEA, Stabilité Génétique Cellules Souches et Radiations, LRTS/iRCM/IBFJ, CEA, 92260 Fontenay-aux-Roses, France; 3 Laboratory of Medical Radiobiology, Institute of Radiological Protection and Nuclear Safety, Fontenay-aux-Roses, France; 4 Université Grenoble Alpes, Inserm, CEA, UMR BioSanté U1292, CNRS, FR2048, CEA, 38000 Grenoble, France; 5 Institut de Recherche Biomédicale des Armées (IRBA), 92141 Clamart, France; 6 UMR-S-MD 1197, Ministère des Armées et Université Paris Saclay, Villejuif, France

**Keywords:** Muse cells, Radio-induced gastro-intestinal syndrome, Stem cell microenvironment, Regeneration

## Abstract

**Background:**

Human multilineage-differentiating stress enduring (Muse) cells are nontumorigenic endogenous pluripotent-like stem cells that can be easily obtained from various adult or fetal tissues. Regenerative effects of Muse cells have been shown in some disease models. Muse cells specifically home in damaged tissues where they exert pleiotropic effects. Exposition of the small intestine to high doses of irradiation (IR) delivered after radiotherapy or nuclear accident results in a lethal gastrointestinal syndrome (GIS) characterized by acute loss of intestinal stem cells, impaired epithelial regeneration and subsequent loss of the mucosal barrier resulting in sepsis and death. To date, there is no effective medical treatment for GIS. Here, we investigate whether Muse cells can prevent lethal GIS and study how they act on intestinal stem cell microenvironment to promote intestinal regeneration.

**Methods:**

Human Muse cells from Wharton’s jelly matrix of umbilical cord (WJ-Muse) were sorted by flow cytometry using the SSEA-3 marker, characterized and compared to bone-marrow derived Muse cells (BM-Muse). Under gas anesthesia, GIS mice were treated or not through an intravenous retro-orbital injection of 50,000 WJ-Muse, freshly isolated or cryopreserved, shortly after an 18 Gy-abdominal IR. No immunosuppressant was delivered to the mice. Mice were euthanized either 24 h post-IR to assess early small intestine tissue response, or 7 days post-IR to assess any regenerative response. Mouse survival, histological stainings, apoptosis and cell proliferation were studied and measurement of cytokines, recruitment of immune cells and barrier functional assay were performed.

**Results:**

Injection of WJ-Muse shortly after abdominal IR highly improved mouse survival as a result of a rapid regeneration of intestinal epithelium with the rescue of the impaired epithelial barrier. In small intestine of Muse-treated mice, an early enhanced secretion of IL-6 and MCP-1 cytokines was observed associated with (1) recruitment of monocytes/M2-like macrophages and (2) proliferation of Paneth cells through activation of the IL-6/Stat3 pathway.

**Conclusion:**

Our findings indicate that a single injection of a small quantity of WJ-Muse may be a new and easy therapeutic strategy for treating lethal GIS.

**Supplementary Information:**

The online version contains supplementary material available at 10.1186/s13287-023-03425-1.

## Introduction

The small intestine is the most radiosensitive tissue of the intra-abdominal area as it is a renewable tissue, especially intestinal mucosa which is replaced every 3–5 days [1]. The intestinal mucosa is divided into three distinct layers including *lamina epithelialis* (LE), *lamina propria* (LP), rich in vascular, lymphatic network and leucocytes, and *muscularis mucosae*. The central component of intestinal mucosal barrier is the LE, organized in two connected structures called villi and crypts. Villi are made up of mature functional cells including mostly absorptive enterocytes connected by tight junctions [2]. Crypts contain two populations of intestinal stem cells (ISC), identified by the markers Lgr5 or Bmi-1, and transit amplifying cells. Lgr5^+^ ISC are mitotically active and ensure the continual renewal of the epithelium cells, whereas Bmi-1^+^ ISC are quiescent and their function during homeostasis or injury is still debate [3, 4]. Regulation of ISC behaviors in intestinal homeostasis occurs within a microenvironment confined to the crypt base, known as the stem cell niche [5]. The microenvironment includes multiple cell types such as Paneth cells, closely tied to Lgr5^+^ ISC and monocytes/macrophages and secretes cell-associated ligands, chemokines, soluble growth factors and cytokines [6, 7]. Intestinal injury is frequently accompanied by inflammation. Paneth cells [8] and macrophages [9] promote the repair of intestinal tissue by regulating ISC function.

Radiation-induced gastrointestinal syndrome (GIS) is a lethal disease occurring after therapeutic or accidental exposure to high doses of whole-body irradiation (IR) or significant whole-abdominal IR [10]. Intestine symptoms occur few days after exposure and their severity depends on the dose of irradiation. They include weight loss and diarrhea, leading to dehydration and electrolyte loss, and to an increased susceptibility to infection due to the intestinal mucosal barrier breakdown, facilitating the entry of bacteria into the bloodstream and leading ultimately to the death by sepsis.

The intestinal clinical signs and symptoms of the GIS result from the lack of replacement of mature functional cells at the surface of the villi, because stem and proliferating cells of the crypts are irreversibly damaged by radiations and die by apoptosis or mitotic death. In addition, IR induces microvascular damages due to endothelial cell apoptosis and an important inflammatory response in the intestine, characterized by inflammatory cell infiltration and an overproduction of pro-inflammatory mediators contributing to perpetuate damage cells [1].

To date, the management of GIS remains confronted with a therapeutic wall. Clinical approaches including cell therapy or administration of different agents have been used to minimize the severity of gastrointestinal injury, but these therapeutic solutions are not routinely used in human [11]. Thus, there is a compelling need for effective and rapid countermeasures.

Multilineage-differentiating stress-enduring (Muse) cells are endogenous pluripotent-like stem cells collectable through the pluripotent stem cell surface marker Stage-Specific Embryonic Antigen 3 (SSEA-3) from multiple sources including the bone marrow, peripheral blood, adipose tissue and umbilical cord [12]. Muse cells can migrate into the injured tissues where they exert pleiotropic effects including anti-inflammatory actions, vascular protection and anti-apoptotic responses [13]. Another important and unique feature is that allogeneic-Muse cells escape host immune-rejection after intravenous administration and survive in the host tissue as differentiated cells for over 6 months without immunosuppressive treatment [14]. Based on the safety and efficacity already demonstrated in preclinical studies, clinical trials using intravenous infusion of Muse cells are currently in progress for the treatment of human diseases such as acute myocardial infarction, ischemic stroke, spinal cord injuries and amyotrophic lateral sclerosis [15, 16].

Here, we show that Muse cells from umbilical cord matrix of Wharton’s jelly promote a beneficial stem cell microenvironment, favoring the reconstitution of intestinal barrier that is a requisite for recovery and survival following GIS.

## Methods

### Cell preparation and culture

Human adult bone marrow Mesenchymal Stem cells (BM-MSC) derived from healthy donors were purchased from Lonza (#PT-2501).

Human fetal MSC (WJ-MSC) were derived from Wharton’s jelly of umbilical cords (UC), obtained from normal full-term deliveries after maternal informed consent at Maternity unit from Bicêtre AP-HP Hospital (Le Kremlin-Bicêtre, France). According to the French law, article L.1243-3 of the Public Health Code, a prior approval by an Institutional Review Board was not required. WJ-MSC were isolated as previously described [17]. Briefly, UC were collected and placed in a transport solution containing phosphate buffer saline (Gibco™ DPBS, #14190144, Fisher scientific) supplemented with 1 mM EDTA (#E177, VWR), 4% ACD-A (Macopharma) and 0.5% of human serum albumin (hSA, Vialebex, LFB France). Then, UC were incubated for 1 h 30 min in an antibiotic/antifungal bath containing NaCl, 1 g/l vancomycin (GSK, UK), 1 g/l Clamoxyl® (GSK, UK), 0.5 g/l amikacine (Mylan, France) and 0.05 g/l fungizone (Bristol Myers Squibb, France) optimized to prevent the development of pathogens linked to the contamination of samples at the time of delivery. Then, UC were cut in 2 cm-long pieces and frozen in a solution of 50% Gibco™ RPMI-1640 (Life Technologies) + 50% glycerol (Sigma-Aldrich) before storage at − 80 °C until WJ-MSC isolation. UC pieces were thawed after a resting period of 30 min at room temperature (RT), cut into smaller pieces around 1–2 mm^3^ and digested for 1 h at 37 °C in a solution of DPBS containing 3 mM CaCl_2_, 300 U/ml collagenase type I (#17100017, Fisher scientific) and 1 mg/ml hyaluronidase (#HX0514, Calbiochem-Merck) and were then placed in a DPBS + 0.025% trypsin–EDTA (#R001100, Fisher scientific) for 30 min at 37 °C. After filtration through a 100 µm cell strainer and centrifugation at 200*g* for 10 min, cells were seeded at a 12,000 cells/cm^2^ density in a MEMα medium (#01-042-1, Biological Industries) supplemented with 0.01 mg/ml ciprofloxacine (Panpharma, France), 2 U/ml heparin (Choay, France) and 5% platelet lysate (obtained from platelet apheresis collection performed at the ‘‘Centre de Transfusion Sanguine des Armées’’, Clamart, France). WJ-MSC were amplified until passage 2 and frozen in MEMα supplemented with 10% hSA and 10% dimethylsulfoxide (Sigma-Aldrich) for storage at − 80 °C.

For Muse cell isolation, BM- or WJ-MSC were cultured at a 15,000 cells/cm^2^ density in a Gibco™ Low-glucose DMEM + GlutaMAX medium (#11570586, Fisher scientific) with 10% fetal bovine serum (FBS, #SH30071.03IH, HyClone™, Fisher scientific), 1 ng/ml human FGF-2 (#130-093-564, Miltenyi Biotec) and 0.1 mg/ml Gibco™ kanamycin sulfate (#11578876, Fisher scientific) at 37 °C in 95% air and 5% CO_2_. Cells from passage 7 were used for multilineage-differentiating stress-enduring (Muse) cell isolation. GFP-labeled WJ-MSC were generated using lentiviral plasmid pTrip-MND-GFP.

### Mass spectrometry-based proteomic analyses

Three biological replicates of WJ-Muse and BM-Muse cells were analyzed. The proteins were directly solubilized in Laemmli buffer, heated for 10 min at 95 °C and stacked in the top of a 4–12% NuPAGE gel (Invitrogen). After staining with R-250 Coomassie Blue (Biorad), proteins were digested in-gel using trypsin (modified, sequencing purity, Promega), as previously described [18]. The resulting peptides were fractionated by tip-based strong cation exchange (3 M Empore). For this, peptides were dissolved in 5% acetonitrile (ACN), 1% trifluoroacetic acid and eluted in 4 fractions (F1: 100 mM ammonium acetate (AA), 20% ACN, 0.5% formic acid (FA); F2: 175 mM AA, 20% ACN, 0.5% FA; F3: 375 mM AA, 20% ACN, 0.5% FA; F4: 80% ACN, 5% ammonium hydroxide) before desalting using C18 reverse phase chromatography (Ultra-Micro SpinColumns, Harvard Apparatus). NanoLC-MS/MS analyses of peptides eluted in each fraction were performed using an Ultimate 3000 RSLCnano coupled to a Q-Exactive HF (Thermo Fisher Scientific) using a 240-min gradient. For this purpose, the peptides were sampled on a precolumn (300 μm × 5 mm PepMap C18, Thermo Scientific) and separated in a 75 μm × 250 mm C18 column (Reprosil-Pur 120 C18-AQ, 1.9 μm, Dr. Maisch). The MS and MS/MS data were acquired by Xcalibur (Thermo Fisher Scientific).

Peptides and proteins were identified by Mascot (version 2.6.0, Matrix Science) through concomitant searches against the Uniprot database (*Homo sapiens* taxonomy, October 2019 version), a homemade database containing the sequences of classical contaminant proteins found in proteomic analyses (bovine albumin, keratins, trypsin, etc.), and the corresponding reversed databases. Trypsin/P was chosen as the enzyme, and two missed cleavages were allowed. Precursor and fragment mass error tolerances were set, respectively, at 10 ppm and 25 mmu. Peptide modifications allowed during the search were: Carbamidomethyl (C, fixed), Acetyl (Protein N-term, variable) and Oxidation (M, variable). The Proline software [19] was used for the compilation, grouping, and filtering of the results (conservation of rank 1 peptides, peptide length ≥ 7 amino acids, peptide-spectrum-match score ≥ 25, false discovery rate of peptide-spectrum-match identifications < 1% as calculated on peptide-spectrum-match scores by employing the reverse database strategy, and minimum of one specific peptide per identified protein group). Proline was then used to perform MS1 label-free quantification of the identified protein groups. The mass spectrometry proteomics data have been deposited to the ProteomeXchange Consortium via the PRIDE [20] partner repository with the dataset identifier PXD036883.

Statistical analysis was then performed using the ProStaR software [21]. Proteins identified in the contaminant database and proteins detected in less than three replicates of one condition were removed. After log2 transformation, abundance values were normalized by median centering, before missing value imputation (slsa algorithm for partially observed values in the condition and DetQuantile algorithm for totally absent values in the condition). Statistical testing was then conducted using limma, whereby differentially expressed proteins were sorted out using a fold change cut-off of 1 and a *p* value cut-off of 0.005, leading to a FDR < 1% according to the Benjamini–Hochberg estimator. Proteins found differentially abundant, but detected in less than three replicates in the condition in which they were found to be more abundant, were manually invalidated (*p* value = 1). Gene Ontology (GO) term enrichment analysis was performed with DAVID Bioinformatics resources.

### SSEA-3 staining for Muse cell isolation

When overconfluent, cells were washed, trypsinized and centrifuged for 5 min at 400*g*. Pellet was resuspended in FluoroBrite™ DMEM (#A1896701, Fisher scientific) for washing and then centrifuged. After discarding the supernatant, cells were resuspended in a freshly prepared cold FACS buffer (FluoroBrite™ DMEM containing 0.5% bovine serum albumin (BSA) and 2 mM EDTA) at a maximal concentration of 1 × 10^6^ cells/100 µl. Cells were incubated on ice for 1 h with anti-SSEA-3 antibody at a 1:250 dilution (mixing gently every 10 min) (#MAB4303-I, Millipore). Cells were then centrifuged and washed 3 times with FACS buffer. A secondary anti-rat IgM antibody conjugated to APC (#112-136-075, Jackson ImmunoResearch Lab.) was added to the cells for 1 h on ice at a 1:100 dilution (mixing gently every 10 min). Cells were centrifuged and washed 3 times with FACS buffer. After filtration through a 30-µm cell strainer, SSEA-3^+^ cells were isolated with a BD FACS Aria II SORP cell sorter (Becton Dickinson) using a 100 µm nozzle. Freshly sorted SSEA-3^+^ (Muse) cells were washed with DPBS, centrifuged and resuspended in sterile DPBS (50,000 cells in 100 µl/mouse) for intravenous (i.v.) injection. GFP-labeled WJ-Muse were isolated as SSEA-3^+^ cells from GFP^+^ WJ-MSC.

### Bulk production of Muse cell derived-clusters by using methylcellulose gel

To avoid cell adherence, plates were pre-coated with a 3% poly-HEMA solution in 95% Ethanol [poly(2-hydroxyethyl methacrylate), #P3932, Sigma-Aldrich]. FACS-sorted Muse cells were mixed to Low-glucose DMEM + GlutaMAX medium containing 10% FBS and 0.9% methylcellulose (MethoCult H4100, #04100, StemCell Technologies). After plating, cells were maintained in culture for 7–10 days by addition of fresh Low-glucose DMEM + GlutaMAX medium containing 10% FBS every 3 days. Then, clusters were picked up for analysis.

### Muse cell cryopreservation

FACS isolated SSEA-3^+^ Muse cells were centrifuged (300*g*, 5 min) immediately after sorting. Supernatant was discarded, and cells were resuspended in 1 ml BAMBANKER™ (#W1W302-14681, Sobioda) and transferred into a cryotube. After a 24-h freezing at − 80 °C (isopropanol freezing container), tubes were stored into liquid nitrogen.

The day of injection, cells were thawed in a water bath at 37 °C and added to 9 ml of pre-warmed FBS. After centrifugation, pellet was resuspended in sterile DPBS (50,000 cells in 100 µl/mouse) for i.v. injection.

### Mouse treatment with human WJ-Muse cells or MSC

At different time post-irradiation, fresh or cryopreserved human MSC (50,000) or Muse cells (50,000) were injected in 100 µl sterile DPBS in the retro-orbital vein of the mouse, that was previously anesthetized under isoflurane (1.5% in oxygen). No immunosuppressant was delivered to the injected mice.

### Evaluation of pluripotency and of differentiation ability of Muse cells by quantitative RT-PCR

Total RNA was extracted with the RNeasy Plus Micro kit (#74034, QIAGEN) and reverse-transcribed with random primers and Superscript IV (#18090050, Invitrogen). Quantitative PCR reactions were performed using the Power SYBR™ green Master mix (#4367659, ThermoFisher Scientific) in a StepOne™ Real-Time PCR System (Applied Biosystems). Primer sequences are listed in Table 1.

**Table 1:** List of human (h) and murine (m) qRT-PCR primers

Genes	Forward primers	Reverse primers
*h-Beta-actin*	5′-CACCATTGGCAATGAGCGGTTC	5′-AGGTCTTTGCGGATGTCCACGT
*h-Sox2*	5′-GTCACAGCATGATGCAGGACCA	5′-TCTGCGAGCTGGTCATGGAGTT
*h-Nanog*	5′-CTCCAACATCCTGAACCTCAGC	5′-CGTCACACCATTGCTATTCTTCG
*h-Oct3/4*	5′-CCTGAAGCAGAAGAGGATCACC	5′-AAAGCGGCAGATGGTCGTTTGG
*h-CK18*	5′-CAAGGAGGAGCTGCTCTTCATG	5′-TGGTGCTCTCCTCAATCTGCTG
*h-OCLN*	5′-CCTATAAATCCACGCCGGTTC	5′-AACGAGGCTGCCTGAAGTCA
*h-IDO*	5′-TCTCATTTCGTGATGGAGACTGC	5′-GTGTCCCGTTCTTGCATTTGC
*h-TGFbeta1*	5′-GGCCAGATCCTGTCCAAGC	5′-GTGGGTTTCCACCATTAGCAC
*h-Cox2*	5′-CAGCCATACAGCAAATCC	5′-ATCCTGTCCGGGTACAAT
*h-PD-L1*	5′-GGACAAGCAGTGACCATCAAG	5′-CCCAGAATTACCAAGTGAGTCCT
*m-GAPDH*	5′-AAGGTGAAGGTCGGAGTCAAC	5′-GGGGTCATTGATGGCAACAATA
*m-Nos2*	5′-GGTCTTTGACGCTCGGAACTGTAG	5′-CACAACTGGGTGAACTCCAAGGTG
*m-Arg1*	5′-GTGAAGAACCCACGGTCTGT	5′-CTGGTTGTCAGGGGAGTGTT
*m-Lgr5*	5′-CCTACTCGAAGACTTACCCAGT	5′-GCATTGGGGTGAATGATAGCA
*m-Lysozyme*	5′-TGACATCACTGCAGCCATAC	5′-TGGGACAGATCTCGGTTTTG
*m-Olfm4*	5′-CAGCCACTTTCCAATTTCACTG	5′-GCTGGACATACTCCTTCACCTTA
*m-Ascl2*	5′-AGGTCAGTCAGCACTTGGCATT	5′-TCCTGGTGGACCTACCTGCTT
*m-Hprt*	5′-GCTGGTGAAAAGGACCTCT	5′-CACAGGACTAGAACACCTGC

### Evaluation of immune privilege of Muse cells

10^4^ isolated human Muse cells were co-cultured with, either 10^5^ Ficoll-isolated human peripheral blood mononuclear cells (hPBMC), or 10^5^ isolated murine splenic lymphocytes (mSL) in Gibco™ RPMI-1640 + GlutaMAX medium (#72400-021, Fisher scientific) containing 1 mM sodium pyruvate (#11360-070, Gibco), 1× MEM Non-Essential Amino Acids solution (NEAA, #11140-035, Gibco), 5 µM 2-mercaptoethanol (#31350010, Gibco), 10% FBS and 100 U/ml Gibco™ penicillin streptomycin (#15140122, Fisher scientific). Six days later, 10 µM 5-bromo-2′-deoxyuridine (BrdU) was added to the medium for 2 h and cells were collected to measure BrdU incorporation in CD3^+^ lymphocytes by flow cytometry. As positive controls of proliferation, hPBMC or mSL were treated with 5 µM of concanavalin A (#C5275, Sigma-Aldrich). Briefly, cells were stained with anti-human or anti-mouse CD3 antibody for 15 min at 4 °C. After washing, cells were fixed and permeabilized with the BD Cytofix/Cytoperm™ kit (#554722, BD Biosciences) following the manufacturer’s protocol. After a DNAse I treatment for 1 h at 37 °C, cells were incubated with a FITC anti-BrdU antibody for 30 min at RT. After washing and centrifugation, cells were resuspended in DPBS and analyzed by FACS.

### Evaluation of the immunosuppressive potential (allogeneic and xenogeneic) of Muse cells

A total of 5000 or 10,000 or 20,000 isolated human Muse cells were cultured in 96-well plates at 37 °C, in the presence or not of 15 ng/ml TNFα and 10 ng/ml IFNγ. After 48 h, Muse cells were washed with DPBS before co-culturing them with 100,000 hPBMC or mSL in Gibco™ RPMI-1640 + GlutaMAX medium containing 1 mM sodium pyruvate, 1× NEAA, 5 µM 2-mercaptoethanol, 10% FBS and 100 U/ml Gibco™ penicillin streptomycin for a 2 h-contact. Then, 5 µM concanavalin A was added to the medium, and after 6 days of culture, cells were incubated for 2 h with 10 µM BrdU and collected to measure BrdU incorporation in CD3^+^ T-lymphocytes by flow cytometry.

### In vitro differentiation of Muse cells

Multipotency of Muse cell was assessed by testing their ability to differentiate into adipocytes, osteoblasts and epithelial cells in the presence of specific differentiation media.

Isolated cells were cultured overnight at a 15,000 cells/cm^2^ density in a Gibco™ Low-glucose DMEM + GlutaMAX medium with 10% FBS, 1 ng/ml human FGF-2 and 0.1 mg/ml Gibco™ kanamycin sulfate, at 37 °C in 95% air and 5% CO_2_.

#### Epithelial differentiation

10 µM retinoic acid (#R2625, Sigma-Aldrich) was added to the medium, which was replaced every 2–3 days. After 4 weeks, cells were washed with DPBS and placed in RLT buffer to extract RNA for qRT-PCR.

#### Osteogenic differentiation

DMEM medium was replaced by a StemXVivo Osteogenic/Adipogenic Base Media (#CCM007, R&D Systems) with 1% kanamycin sulfate and 5% StemXVivo human osteogenic supplement 20× (#CCM008, R&D Systems), which was replaced every 2–3 days. After 2 weeks, cells were washed with DPBS and fixed with 4% paraformaldehyde for immunological (osteocalcin) staining.

#### Adipogenic differentiation

DMEM medium was replaced by a StemXVivo Osteogenic/Adipogenic Base Media (#CCM007, R&D Systems) with 1% kanamycin sulfate and 1% StemXVivo human adipogenic supplement 100× (#CCM0011, R&D Systems), which was replaced every 2–3 days. After 3 weeks, cells were washed with DPBS and fixed with 4% paraformaldehyde for histological (Oil red O) staining.

### Co-culturing of BMDM and WJ-Muse/MSC

BMDM were isolated using standard protocols [22]. Primary macrophages were derived from murine bone marrow cells and were cultured alone or in the presence of 50,000 WJ-Muse or 50,000 MSC (ratio 1:1) in IMDM supplemented with 10% FBS, 1% penicillin streptomycin, 10 mM 1-thioglycerol (#M1753, Sigma-Aldrich) and 25 ng/ml mouse M-CSF (#130-101-706, Miltenyi Biotec). After 7 days of culture, BMDM were collected in RLT buffer for RNA extraction.

### Mice

For this study, 319 8-week-old C57BL/6JRj male mice, weighting 22–26 g, were purchased from Janvier Laboratory (Le Genest Saint Isle, France) and were housed in the IRSN specific-pathogen-free-animal facility, accredited by the French Ministry of Agriculture to perform experiments on rodents. Acclimatization of mice in the animal facility was at least 1 week.

The manuscript adheres to the ARRIVE guidelines for the reporting of animal experiments.

All experiment procedures were approved and performed in accordance with the guidelines of the French Ministry of Agriculture’s Animal Ethics Committee (EC Directive 2010/63/EU and French Decree 2013–118). The use of animals has been approved by an IRSN ethics committee (CEEA 546 Number 81 for IRSN unit). The approved project's title is: Evaluation of the therapeutic efficacy of MUSE cells in the management of radio-induced gastrointestinal syndrome in mice; IRSN project P18-11, Approval number: APAFIS#17492-2018111211126943-v1, 01/10/2019. In this study, experimental protocol was in line with standard support treatment recommended for patient presenting an acute radiation syndrome [23].

Thus, mice received antibiotics in drinking water (8 g/l Avemix®) during the whole study.

The animals were housed by four to a cage, with access to food and water ad libitum and with light and dark cycles. Every effort was made to minimize suffering by adding enrichment such as wooden sticks and tunnels. At the reception of the animals, traceability and individual identification of animals were achieved using microchip implants. Animals were randomly distributed in the cages of the different groups. The experimenters were aware during the allocation, the conduct of the experiment, the outcome assessment, and the data analysis. All experiments were performed under gaseous anesthesia with isoflurane (Aerrane, 104 Baxter SA, Lessines, Belgium) before cervical dislocation**.** To evaluate the treatment using Muse cells, the mouse experimental groups were then divided into 3 groups of 6 mice each: non-irradiated mice, untreated irradiated mice and Muse-treated irradiated mice. We used the NC3Rs site (eda.nc3rs.org.uk/experimental-design-group#PowerCalc) to determine the necessary and sufficient number of mice per group to obtain statistically significant results.

### Irradiation and treatment

Mice were irradiated under continuous anesthesia (1.5% isoflurane in oxygen) with a medical linear accelerator (Elekta Synergy) delivering 4MVp X-rays. Reference dosimetry measurements were performed using a 0.125 cm^3^ cylindrical ionization chamber, calibrated in dose to water in a mouse equivalent tissue phantom placed on a Plexiglas support. A localized 2-cm-large abdominal irradiation window containing intestine was determined to avoid an exposure of the upper thorax and of extremities. Mice were irradiated at 18 Gy with a 2.5 Gy/min dose-rate.

### Histology and immunostaining

Mice were euthanized by cervical dislocation at days 1, 3.5, 7 and 30 post-irradiation. Freshly isolated ileal tissue was excised, flushed with cold DPBS and a piece of 1 cm-long was fixed in 4% paraformaldehyde before embedding in paraffin., 5-µm-thick sections were de-paraffinized, rehydrated and stained with a hematoxylin/eosin/safran (HES) coloration.

For immunofluorescent staining, a pretreatment method using antigen retrieval with pH 6-citrate buffer (#ZUC028-500, Zytomed systems) was used. Sections were then permeabilized for 10 min at RT with 0.1% Triton X-100 in DPBS containing calcium and magnesium (DPBS Ca^++^ Mg^++^ #14040091, Fisher scientific) and the non-specific binding was blocked for 30 min with a solution of 5% normal goat serum (NGS) and 1% BSA in DPBS Ca^++^ Mg^++^. Sections were then incubated overnight at 4 °C with primary antibodies listed in Table 2. After washing, sections were probed with appropriate fluorescent-conjugated secondary antibodies for 45 min at RT and cell nuclei were stained for 5 min with 1 µg/ml DAPI.

**Table 2:** List of antibodies used in immunohistology

Antigen	Clone	Catalog #	Supplier
Osteocalcin	190125	MAB1419	R&D systems
Lysozyme	EPR2994	Ab108508	Abcam
Ki67	–	Ab15580	Abcam
CD24	M1/69	Ab64064	Abcam
CD68	–	Ab125212	Abcam
CD206	–	AF2535	R&D systems
GFP	–	Ab6662	Abcam
ZO-1	–	40-2200	Thermo Fisher scientific

ZO-1 immunohistochemical staining was performed on a VENTANA BenchMark Ultra automated staining instrument (Ventana Medical Systems) according to the manufacturer’s protocol [24]. Slides were counterstained with hematoxylin.

### In situ apoptosis detection

Apoptosis was detected by using the TUNEL assay (in situ Cell Death Detection Kit, Fluorescein, #11684795910, Roche Diagnostics, France) following the manufacturer’s protocol. Briefly, after deparaffinization and rehydration, intestinal tissue sections were pre-treated with Proteinase K and incubated for 1 h at 37 °C with a mixture of Terminal deoxynucleotidyl Transferase (TdT) enzyme and fluorescein-labeled nucleotide polymers in a cacodylate buffer. Sections were then washed with a solution of 0.05% Tween-20 in DPBS and mounted with a Vectashield medium containing DAPI (#H-1200, Vector).

### Intestinal permeability

In vivo intestinal permeability was measured in mice at day 7 after irradiation, by administrating FITC-Dextran (#46944, Sigma-Aldrich) by gavage 4 h before euthanasia (0.6 mg/g body weight). Blood was harvested by cardiac puncture. Standard curves were obtained by diluting the FITC-dextran in DPBS. The concentration of FITC-Dextran in plasma of differently treated groups of mice was measured with a microplate Luminometer (Mithras LB940, Berthold) at a 485 nm excitation and a 520 nm emission.

### Cytokine quantification in intestine supernatant

A piece of 600–700 µg of terminal ileum was placed in a Gibco™ RPMI-1640 + GlutaMAX medium (#61870036, Fisher scientific) containing 100 U/ml Gibco™ penicillin streptomycin (#15140122, Fisher scientific) and incubated for 7 h at 37 °C in 95% air and 5% CO_2_. Supernatant was then collected, aliquoted after addition of protease inhibitors and stored at − 80 °C before use.

Levels of inflammatory cytokines were measured with the BD Cytometric Bead Array (CBA). Mouse Inflammation Kit (#552364, BD Biosciences), following the manufacturer’s protocol. A FACS Canto II flow cytometer was used for sample acquisition.

### Isolation of Paneth cells from intestinal lamina epithelialis (LE)

To isolate Paneth cells from intestinal LE, the terminal ileum of mice was excised and washed with cold DPBS. After measurement of length and weight, gut was opened longitudinally, cut into 0.5-cm pieces and incubated for 20 min at 37 °C under agitation (25 rpm) in a pre-digestion buffer containing 10 mM Hepes, 5 mM EDTA, 5% FBS and 1 mM DTT in Hank’s Balanced Salt Solution (HBSS) without Ca^++^ Mg^++^ (#14190, Fisher scientific). After 10-s vortexing, cell solution was filtered (100 µm) and stored. Tissue pieces were a second time incubated for 20 min at 37 °C under agitation in a fresh pre-digestion buffer. After 10-s vortexing, cell solution was filtered and mixed to the previous stored cell suspension. After centrifugation, cells were counted (Kova slide) and incubated with FACS antibodies listed in Table 3 and a viability marker. Cell isolation was performed with a BD FACSAria™ II cell sorter (BD Biosciences). Paneth cells were defined as: CD45^neg^, EpCAM^+^, CD44^+^, CD24^+^, CD166^low^ [25].

**Table 3:** List of antibodies used in flow cytometry

Antigen	Conjugate	Clone	Catalog #	Supplier
SSEA-3	–	MC-631	MAB4303-I	Merck Millipore
BrdU	FITC	B44	347583	BD Biosciences
Human CD3	PercP-Cy5.5	SK7	981008	BioLegend
Mouse CD3	PerCP	145-2C11	100328	BioLegend
CD45	PE/Cy7	30-F11	103114	BioLegend
CD44	FITC	IM7	103005	BioLegend
CD90	Brilliant Violet 421™	5E10	328122	BioLegend
CD73	PercP-Cy5.5	TY/11.8	127214	BioLegend
HLA-DR	PercP-Cy5.5	Tü39	361710	BioLegend
CD105	PE	MJ7/18	12-1051-82	eBioscience
EpCAM (CD326)	APC	G8.8	118214	BioLegend
CD166	PE	eBioALC48	12-1661-82	eBioscience
CD24	Brilliant Violet 421™	M1/69	101826	BioLegend
CD11b	PE	M1/70	553311	BD Biosciences
Ly6C	APC	HK1.4	17593282	BioLegend
Ly6G	PerCP	1A8	127616	BioLegend

### Capillary electrophoresis immunoassay (simple western)

Total proteins from intestinal *lamina epithelialis* or from isolated Paneth cells were extracted with a RIPA buffer containing a cocktail of 1X protease inhibitors (#11836145001, Roche) and 1× phosphatase inhibitors (#P2850; #P5726, Sigma-Aldrich).

After 5-min denaturation at 95 °C in the presence of 5× Fluorescent Master Mix (PS-FL01-8, ProteinSimple), samples were assayed on a ProteinSimple Wes automated capillary-based electrophoresis instrument with Wes Separation Module protocol (ProteinSimple). The 12–230 kDa Separation Module 8 × 25 capillary cartridges (SM-W004, ProteinSimple) and the Anti-Rabbit Detection Module (DM-001, ProteinSimple) were used. Proteins were identified using rabbit primary antibodies listed in Table 4. Results (peak area) were analyzed using Compass for SW software v5.0.1.

**Table 4:** List of antibodies used in simple western

Antigen	Clone	Catalog #	Supplier
Phospho-Stat3 (Tyr705)	D3A7	9145	Cell signaling technology
Stat3	79D7	4904	Cell signaling technology
Cleaved caspase-3	5A1E	9664	Cell signaling technology
Lysozyme	EPR2994	Ab108508	Abcam
GAPDH	14C10	2118	Cell signaling technology

## Results

### Comparative characterization of human Muse cells (Muse) purified from Warton’s jelly and bone marrow

**Fig. 1:**
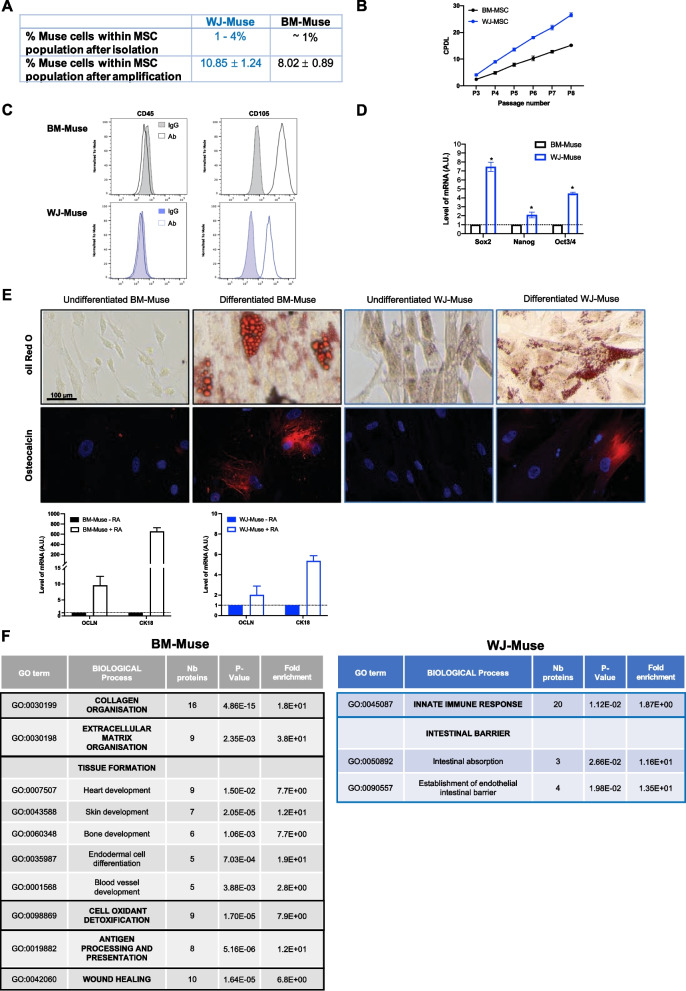
WJ-Muse have biological advantages in comparison with BM-Muse. **A** Proportion of Muse cells among the BM-MSC or the WJ-MSC population, before and after amplification until passage 7 of culture. **B** Comparison of the cumulative population doubling levels (CPDL) of BM-MSC and WJ-MSC between passage 3 and passage 8 of culture. Data are represented with means ± SEM. **C** Representative flow analysis of CD45 (hematopoietic marker) and CD105 (MSC marker) in BM-Muse and WJ-Muse cells. **D** mRNA expression by RT-qPCR of *Sox2*, *Nanog* and *Oct3/4* in WJ-Muse compared to BM-Muse. Data are represented with means ± SEM, *p < 0.05 (two-tailed Mann–Whitney *U* test). **E** Adipogenic, osteogenic and epithelial differentiation of BM-Muse and WJ-Muse. Upper images: morphological features of BM-Muse and WJ-Muse are illustrated before and after differentiation. Adipocyte cells are stained with oil Red O; osteocyte cells are immunostained with osteocalcin (red) and counterstained with Dapi (blue); Lower histograms: epithelial differentiation was shown in BM-Muse and WJ-Muse with mRNA expression of *cytokeratin18* (CK18) and *occluding* (OCLN) with or without retinoic acid treatment. **F** Comparative analysis of biological processes occurring in BM-Muse and WJ-Muse cells, identified by proteomic analysis

Previous reports indicate that several human tissues are readily accessible for Muse isolation, including adult tissues such as bone marrow (BM) and embryonic tissues such as Wharton’s jelly (WJ) of umbilical cord [26, 27]. We first compared WJ-Muse derived from mesenchymal stem cells (MSC) isolated from WJ of umbilical cord by enzymatic digestion and BM-Muse obtained from marketed MSC to select the best source of Muse with a potential therapeutic use in GIS. Almost 100% of isolated WJ-MSC are viable, negative for the hematopoietic surface marker CD45 and positive for different mesenchymal surface markers such as CD44, CD105, CD90 and CD73 (Additional file 1: Fig. S1A). Based on the Muse specific SSEA-3 marker, the mean percentage of Muse obtained after MSC isolation from Wharton’s jelly ranged from 1 to 4%, while the mean percentage of Muse within BM-derived MSC was about 1% (Fig. 1A). To get a high number of cells, Muse were isolated by flow cytometry after in vitro amplification of WJ-MSC or BM-MSC using culture protocols previously described [28] (Additional file 1: Fig. S1B). In culture, WJ-MSC exhibited a higher proliferative capacity than BM-MSC, with higher cumulative cell population (Fig. 1B). After seven passages of culture of MSC, the mean percentage of WJ-Muse was 11%, while the mean percentage of BM-Muse was 8% (Fig. 1A and Additional file 1: Fig. S1B). WJ- and BM-Muse share the same characteristics as MSC, (1) the plastic adherence potential and spindle shape fibroblast-like morphology when maintained in standard culture conditions and (2) formed spontaneously clusters in cell suspension culture (Additional file 1: Fig. S1C). Both WJ- and BM-Muse were negative for CD45 and positive for CD105 (Fig. 1C). WJ- and BM-Muse pluripotent potential was studied by RT-qPCR. WJ-Muse expressed higher levels of Sox2, Nanog and Oct3/4 than BM-Muse suggesting a more immature status (Fig. 1D). Then, multilineage differentiation capacity of WJ- and BM-Muse was studied. WJ- and BM-Muse displayed the same ability to differentiate in adipocytes (positive staining of cytoplasmic lipid droplets with oil Red O) (Fig. 1E, top photos), in osteoclasts (positive expression of osteocalcin protein) (Fig. 1E, down photos) and in epithelial cells (expression of *cytokeratin 18* (CK18) and *Occludin* (OCLN)) (Fig. 1E, lower panels). We then characterized the protein repertoires of WJ-Muse and BM-Muse using mass spectrometry (MS)-based quantitative proteomics as WJ- and BM-Muse might express different set of proteins which may influence their biological properties. Among the 6058 reliably identified and quantified human proteins, 744 were differentially expressed, with 343 and 401 proteins found to be significantly more abundant in BM- and WJ-Muse, respectively (Additional file 1: Fig. S1D). Gene Ontology (GO) analysis highlighted common biological properties of BM- and WJ-Muse such as angiogenesis, cell adhesion, cell migration and response to drug (Additional file 1: Fig. S1E). However, proteins enriched in BM-Muse are related to collagen, extracellular matrix, development of tissues, anti-oxidant activity and adaptive immunity, which are important for wound healing and indicated the potential of BM-Muse in tissue damage repair (Fig. 1F, left panel). In contrast, WJ-Muse present proteins involved in innate immune response, which is considered as the first line of host defense in tissue injury. Interestingly, WJ-Muse are also enriched in proteins that are essential for intestinal barrier function such as the ICAM-1 protein, known to regulate the homing and the immunomodulatory activity as observed in intestinal mucosal wound healing (Fig. 1F, right panel) [29].

Altogether, these results showed that WJ-Muse share similar biological characteristics with BM-Muse but present many advantages: (1) a higher frequency, (2) a more immature status, (3) an expression of proteins that interplay with the innate immune response that can modulate the healing process and (4) an expression of proteins involved in intestinal barrier regeneration. Thus, WJ-Muse were good candidates for cell therapy in the GIS.

### Immunoreactivity of WJ-Muse

**Fig. 2:**
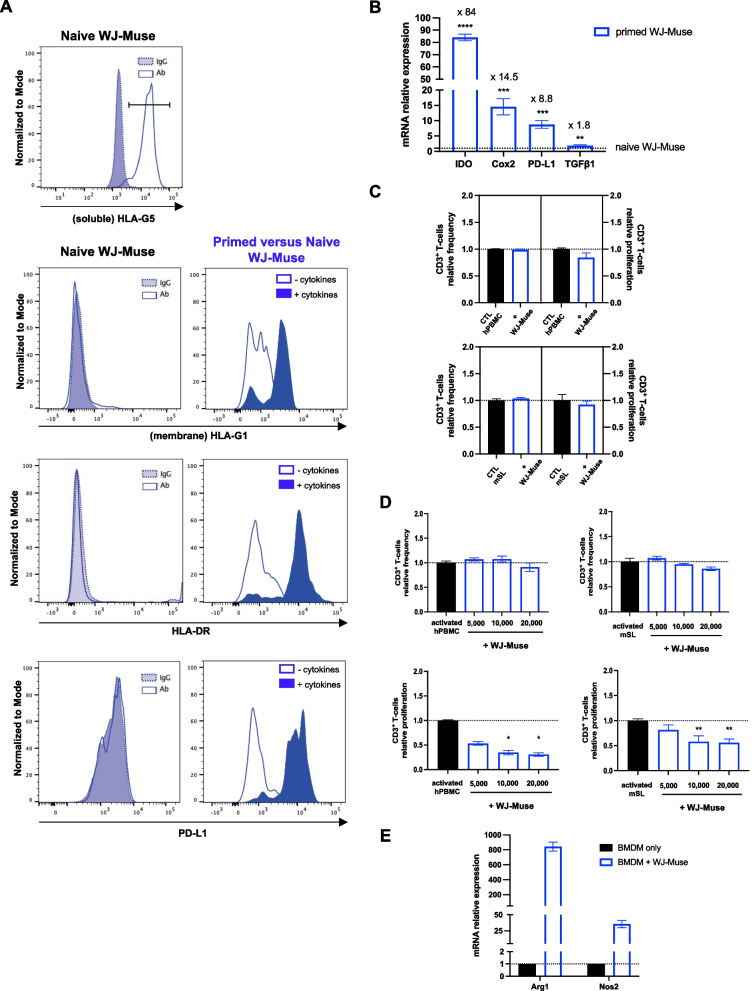
WJ-Muse display immunosuppressive properties. **A** Left: Representative flow analysis of HLA-G5 (soluble human leukocyte antigen-G5 marker), HLA-G1 (membrane-bound human leukocyte antigen-G1 marker), HLA-DR (MHC Class II, human leukocyte antigen marker) and PD-L1 (human Programmed death-ligand 1) in naive WJ-Muse. IgG were used as reference controls. Right: Representative flow analysis of HLA-G1, HLA-DR and PD-L1 in TNFα/IFNγ-primed WJ-Muse compared to naive WJ-Muse. **B** Evaluation of IDO, Cox2, PD-L1 and TGFβ1 expression level by quantitative real time PCR, in TNFα/IFNγ-primed WJ-Muse compared to naive WJ-Muse. Data are represented with means ± SEM, **p < 0.01; ***p < 0.001; ****p < 0.0001 (unpaired Student’s *t* test). **C** 10^5^ human peripheral blood mononuclear cells (hPBMC) or murine spleen lymphocytes (mSL) were co-cultured with 10^4^ WJ-Muse in order to evaluate their allogeneic or xenogeneic immune privilege. Frequency and proliferation of BrdU-labeled CD3^+^ T-cells were analyzed by flow cytometry and compared to hPBMC or mSL alone. Data are represented with means ± SEM. **D** Human peripheral blood mononuclear cells (hPBMC) or murine spleen lymphocytes (mSL) activated with concanavalin A were co-cultured with 5 × 10^3^, 10 × 10^3^ or 20 × 10^3^ WJ-Muse to determine their allogeneic and xenogeneic immunosuppressive potential, respectively. Frequency (top) and proliferation (bottom) of human (left) or murine (right) BrdU-labeled CD3^+^ T-cells were analyzed by flow cytometry and compared, respectively, with activated hPBMC or mSL alone. Data are represented with means ± SEM, *p < 0.05, **p < 0.01 (two-tailed Mann–Whitney *U* test). **E** Arginase-1 (Arg1) and Nitric oxide synthase-2 (Nos2) mRNA expression were measured in bone marrow-derived macrophages (BMDM), which have been co-cultured for 7 days with WJ-Muse. Data are represented with mean ± SEM

Muse isolated from adult tissues have immunosuppressive capacities [30, 31], but no data are currently available from fetal Muse. Thus, we investigated whether WJ-Muse might regulate an immune response by cell membrane expression and/or secretion of immunosuppressive factors. WJ-Muse constitutively expressed soluble HLA-G5 and expressed HLA-G1, HLA-DR and PD-L1 only when WJ-Muse are pre-activated (primed) with the IFNγ and TNFα pro-inflammatory cytokines (Fig. 2A). Compared to naive cells, these pre-activated WJ-Muse over-expressed immunosuppressive factors such as IDO (84-fold), Cox2 (14.5-fold), PD-L1 (8.8-fold) and at a lower level TGFβ1 (1.8-fold) (Fig. 2B). Then, we investigated whether WJ-Muse could regulate the CD3^+^ T-cell responses. Allogeneic co-cultures of WJ-Muse with unstimulated human peripheral blood mononuclear cells (CTL hPBMC) showed no change in the frequency or proliferation of human CD3^+^ T-cells (Fig. 2C, upper panels). Xenogeneic co-cultures with unstimulated murine spleen lymphocytes (CTL mSL) did not modify the frequency and proliferation of murine CD3^+^ T-cells (Fig. 2C, left and right lower panels). Finally, we studied if WJ-Muse could modulate the proliferation of concanavalin A-stimulated hPBMC or mSL. In allogeneic and xenogeneic conditions, the frequency of CD3^+^ T-cells did not change in the presence of different concentrations of WJ-Muse (Fig. 2D left and right upper panels), whereas CD3^+^ T-cell proliferation was significantly reduced when co-cultured with 10,000 or 20,000 WJ-Muse compared to concanavalin A-stimulated hPBMC or mSL alone (Fig. 2D, left and right lower panels).

Finally, as the immunomodulatory capacities of WJ-Muse could extend to other immune cell populations, we studied the effect of WJ-Muse on macrophages. Murine bone marrow-derived macrophages (BMDM) were co-cultured in the absence or presence of WJ-Muse and characterized for M1- (Nos2) or M2- (Arg1) macrophage markers. Co-culture of BMDM with WJ-Muse highly increased the expression of Arg1 (843-fold) and weakly increased the expression of Nos2 (35-fold) (Fig. 2E), indicating that WJ-Muse might drive macrophage polarization toward anti-inflammatory M2-like phenotype, known to be involved in tissue remodeling and repair [32, 33].

Altogether, these results showed that WJ-Muse display immunosuppressive properties through cell contact and/or soluble mediators, which are expressed either constitutively or induced by inflammation. These immunosuppressive properties might regulate their integration in injured tissue after intravenous injection [34] and lead to inhibition of inflammation responses and to stimulation of the regenerative process.

### WJ-Muse migrate in the irradiated intestine and improve survival, prevent weight loss and reduce damages of the small intestine in a GIS mouse model

**Fig. 3:**
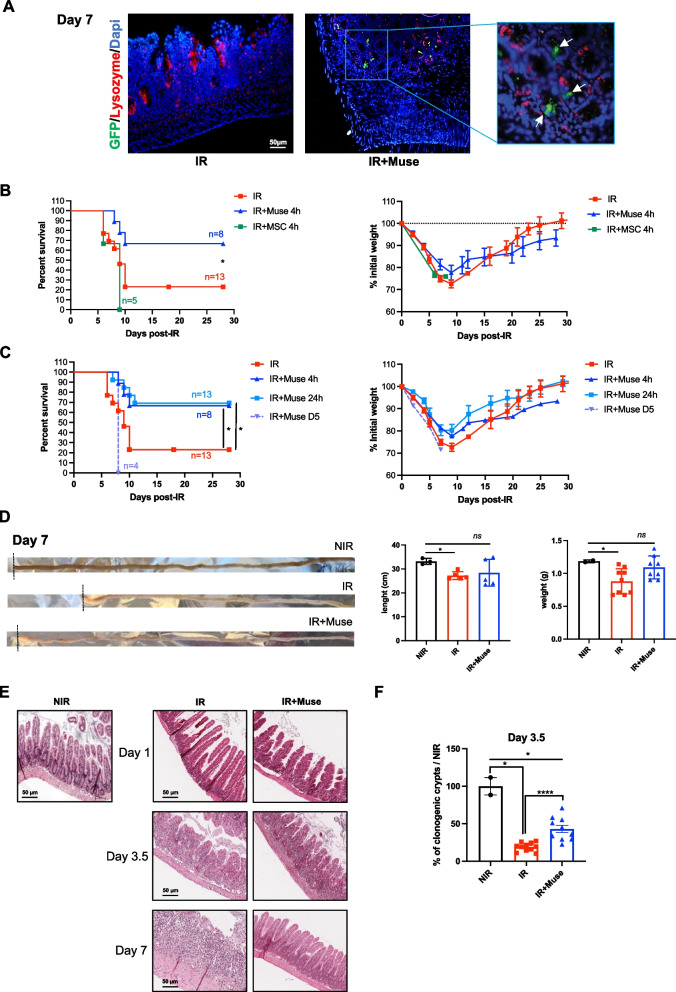
Muse improve survival by maintaining the intestinal integrity in GIS mouse model. **A** Representative images of immunofluorescence showing Lysozyme-positive cells (red) in the small intestine of non-treated or Muse-treated mice at 7 days after irradiation. Integrated GFP^+^ Muse (green) are identified with white arrows. Nuclei were counterstained with DAPI (blue). **B** Kaplan–Meier survival analysis (left) and weight loss changes (right) for 29 days of 18 Gy abdominal exposed mice receiving or not a 50,000 MSC or 50,000 Muse-treatment 4 h after irradiation. Statistical difference of survival between groups was determined by Log-rank (Mantel-Cox) test, with *p ≤ 0.05 considered as significant. Weight data are represented with means ± SEM. **C** Kaplan–Meier survival analysis (left) and weight loss changes (right) for 29 days of 18 Gy abdominal exposed mice receiving or not a 50,000 Muse-treatment 4 h, 24 h or 5 days after irradiation. Statistical difference of survival between groups was determined by Log-rank (Mantel-Cox) test, with *p ≤ 0.05 considered as significant. Weight data are represented with means ± SEM. **D** Illustration (left), length (middle) and weight (right) of the small intestine of non-treated or Muse-treated mice 7 days after irradiation, compared to non-irradiated (NIR) control mice. Data are represented with means ± SEM, *p < 0.05; *ns*: not significant (two-tailed Mann–Whitney U test). **E** Representative HE staining of small intestine of non-treated or Muse-treated mice at 1, 3.5 and 7 days after irradiation, compared to non-irradiated control mice. **F** Histogram plots showing the percentage of surviving clonogenic crypts in small intestine of non-treated or Muse-treated mice at 3.5 days after irradiation, compared to non-irradiated control mice. Data are represented with means ± SEM, *p < 0.05; ****p < 0.001 (two-tailed Mann–Whitney U test)

To investigate any therapeutic potential of WJ-Muse (called Muse) in the treatment of GIS, 50,000 Muse or 50,000 MSC were intravenously injected in C57BL/6 mice 4 h after a 18 Gy-abdominal IR, a localized dose known to induce a lethal GIS within 7–10 days [35]. As Muse can migrate and home into injured tissues [36], we first monitored any migration and homing of Muse in the irradiated small intestine. Seven days after injection of GFP-Muse in irradiated mice, we observed GFP^+^ cells in the environment of intestinal crypts (Fig. 3A) indicating that Muse have integrated the injured tissue.

A survival study showed that 70% of Muse-treated mice survived until 29 days, while untreated or MSC-treated mice died within 10 days (80% and 100%, respectively) (Fig. 3B, left panel). Whereas all mice began to lose weight with a nadir around 7–10 days, Muse-treated mice lost less weight and recovered a normal one 29 days after IR (Fig. 3B, right panel). To specify the time-window of animal management to impair the GIS after IR injury, Muse were injected 24 h or 5 days after the 18 Gy-abdominal IR. Seventy percent of mice treated 24 h after irradiation survived with a reduced weight loss (Fig. 3C, right panel), whereas all mice treated 5 days after irradiation died within 8 days (Fig. 3C, left panel), suggesting that the injection of Muse must should be delivered during the first 24 h after IR injury. Toward a therapeutic use, we studied the effectiveness of cryo-preserved Muse. 98.8% of Muse were alive after thawing and remained positive for SSEA-3 (Additional file 1: Fig. S2A). We treated mice 4 h after abdominal IR with 50,000 cryo-preserved Muse and showed the same effect as fresh Muse with a better survival and a reduction in weight loss compared to untreated mice (Additional file 1: Fig. S2B). Thus, most of in vivo experiments were performed with cryo-preserved Muse.

At different times post-IR, surviving mice were sacrificed and the small intestines were harvested for macroscopic and histological evaluation. GIS mice treated 4 h post-IR with 50,000 Muse displayed neither shortening, nor weight loss of the small intestine 7 days after IR (Fig. 3D). In Muse-treated mice, 7 days post-IR, the crypt-villi structures of the small intestine were indistinguishable from that of non-irradiated mice (Fig. 3E), whereas from 3.5 days post-IR, the small intestine of untreated irradiated mice showed a persistent mucosal architecture destruction, including villus denudation and crypt atrophy (Fig. 3E). The ability of the intestinal epithelium to regenerate depends on the number of surviving stem cells 3.5 days after IR [3]. In accordance with the histological results, at 3.5 days after IR, Muse-treated mice had a twofold increase of clonogenic crypt count compared to untreated mice (Fig. 3F).

Altogether, these results suggest that during GIS, Muse injection mitigated the mouse lethality and maintained intestinal integrity with increased survival of clonogenic crypts.

### Muse promote regeneration of the intestinal epithelium

**Fig. 4:**
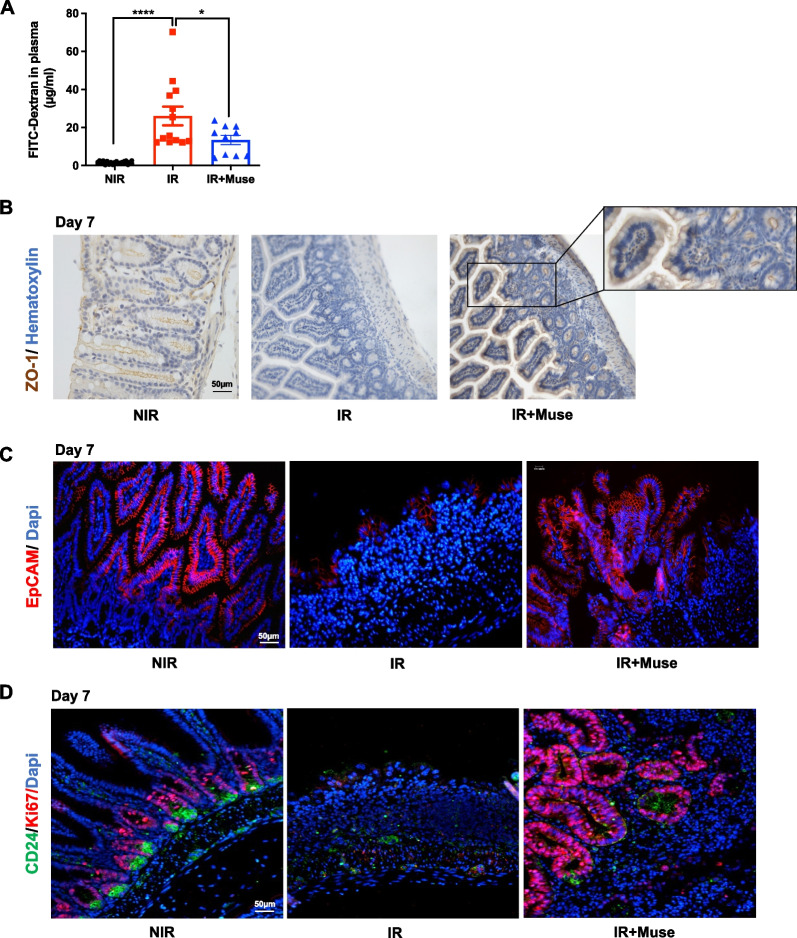
Muse are required for intestinal epithelium regeneration. **A** Intestinal permeability illustrated by histogram plots showing 4-kDa FITC-Dextran levels measured in the plasma of non-treated or Muse-treated mice at 7 days after irradiation, compared to non-irradiated control mice. Data are represented with means ± SEM, *p < 0.05; ****p < 0.001 (two-tailed Mann–Whitney U test). **B** Representative immunohistochemical images showing the expression of ZO-1 protein (brown) in ileum sections of non-treated or Muse-treated mice at 7 days after irradiation, compared to non-irradiated control mice. Nuclei were counterstained with hematoxylin (blue). **C** Representative immunofluorescent images showing EpCAM-positive cells (red) in the small intestine of non-treated or Muse-treated mice at 7 days after irradiation, compared to non-irradiated control mice. Nuclei were counterstained with DAPI (blue). **D** Representative immunofluorescent images showing CD24-positive cells (green) and Ki67-positive cells (red) in the small intestine of non-treated or Muse-treated mice at 7 days after irradiation, compared to non-irradiated control mice. Nuclei were counterstained with DAPI (blue)

High-dose IR disrupts the intestinal epithelial tight junctions and leads to the mucosal barrier dysfunction making it permeable to luminal content [37–39]. To characterize the intestinal permeability 7 days after IR, we quantified the 4-kDa Dextran conjugated with fluorescein isothiocyanate (FITC-Dextran) in the plasma 4 h after gavage of mice. Abdominal IR of untreated mice increased the intestinal permeability compared to non-irradiated mice (26.1 ± 4.9 µg/ml vs. 1.8 ± 0.2 µg/ml). In contrast, Muse treatment restored the permeability of mucosal barrier (13.4 ± 2.4 µg/ml) (Fig. 4A). As the permeability was characterized by tight junction loss, we studied the intestinal integrity by immunohistochemical analyses using zonula occludens-1 (ZO-1) antibody [40]. In untreated irradiated mice, the decreased expression of ZO-1 protein 7 days post-IR indicated a tight junction loss, whereas Muse injection has maintained ZO-1 expression in the cytomembrane of epithelial cells along the villi, as observed in the control non-irradiated mice (Fig. 4B). The maintenance/regeneration of the epithelial barrier is also mediated by epithelial cell adhesion molecules such as EpCAM [41] that modifies cell–cell contact adhesion strength and tissue plasticity and regulates cell proliferation and differentiation [42]. Seven days post-IR, EpCAM protein expression decreased in the small intestine of untreated mice, whereas the EpCAM expression level in the small intestine of Muse-treated mice was similar to the non-irradiated mice (Fig. 4C). Finally, we studied the epithelium regeneration of the small intestine after Muse treatment by Ki67/CD24 double-staining, CD24 being a marker of both intestinal crypt stem cells and Paneth cells [43]. In non-irradiated mice, proliferating epithelial cells (Ki67^+^) were located in the middle of the crypts, whereas they disappeared from the crypts after IR (Fig. 4D, left and middle panels). Muse injection resulted in regeneration of the epithelium via the formation of enlarged hyperproliferative clusters (Fig. 4D, right panel).

Altogether, these results show that, during GIS, Muse injection orchestrated the regenerative process of intestinal epithelium by promoting cellular junction, epithelial adhesion and proliferation of intestinal epithelium cells (IEC).

### Muse increase monocyte recruitment and promote M2-like macrophage polarization in the irradiated intestine

**Fig. 5:**
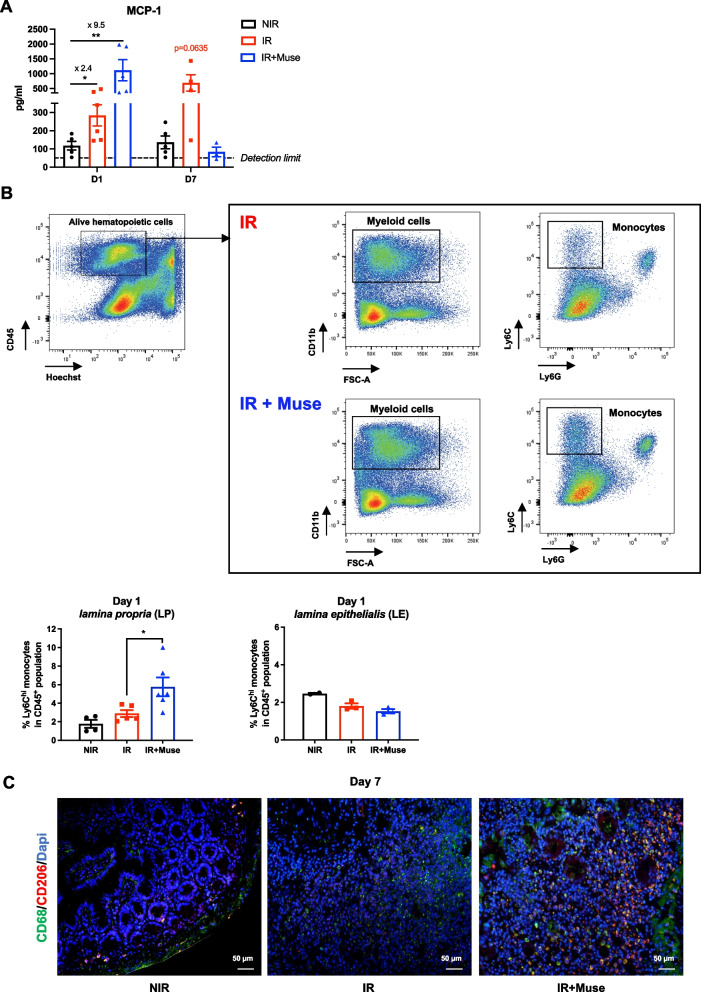
Monocyte/M2-Macrophage recruitment is induced by Muse treatment. **A** Histogram plots showing MCP-1 secretion level in supernatant of 7 h-cultured terminal ileum excised from non-treated or Muse-treated at 1 or 7 days after irradiation, compared to non-irradiated control mice. Data are represented with means ± SEM, *p < 0.05; **p < 0.01 (two-tailed Mann–Whitney U test). **B** Representative flow cytometry gating strategy for analysis of monocyte subset in *lamina propria* isolated from small intestine of non-treated (top) or Muse-treated (bottom) mice at 1 day after irradiation. Histograms plots showing the proportion of M1-like monocytes (Ly6C^hi^) among the alive CD45^+^ population from the *lamina propria* (left) and *lamina epithelialis* (right) fractions of non-treated or Muse-treated mice at 1 day after irradiation, compared to non-irradiated control mice (bottom). Data are represented with means ± SEM, *p < 0.05 (two-tailed Mann–Whitney U test). **C** Representative immunofluorescence images showing CD68-positive (green) and CD206-positive (red) macrophages in the small intestine of non-treated or Muse-treated mice at 7 days after irradiation, compared to non-irradiated control mice. Nuclei were counterstained with DAPI (blue)

The intestinal crypt regeneration after radio-induced injury of small intestine requires the ISC microenvironment including monocytes/macrophages, Paneth cells and molecular mediators [6, 7, 9]. Macrophages are mostly replenished by high turnover from blood monocytes recruited to the sites of injury [44]. First, the protein level of Monocyte-Chemoattractant protein-1 (MCP-1), which enhances the migration of monocytes into tissues during inflammation [45] was studied on small intestine supernatant from untreated or Muse-treated GIS mice compared to non-irradiated mice. One day post-IR, MCP-1 protein level increased 2.4- and 9.5-fold in the untreated and Muse-treated mice, respectively. Seven days post-IR, the MCP-1 protein level returned to normal only in Muse-treated mice (Fig. 5A). This transient higher increase of MCP-1 protein in GIS Muse-treated mice was associated with a twofold increase in Ly6C^hi^ monocytes population in intestinal *lamina propria* from Muse-treated mice, but not in *lamina epithelialis* (Fig. 5B). Finally, Muse treatment led to an increased number of CD68^+^/CD206^+^ double-stained M2-like macrophage population at day 7 post-IR, whereas CD68^+^ macrophages from untreated mice did not express the CD206 marker (Fig. 5C), suggesting that Muse might favor macrophage polarization toward an anti-inflammatory M2 phenotype in injured small intestine.

Altogether, these results suggest that Muse injection could rapidly but transiently enhance MCP-1 production that is associated with an early recruitment of monocytes into the *lamina propria*. Seven days post-IR, Muse treatment is associated with the orientation of macrophages toward a M2 phenotype. These two features might contribute to intestinal tissue regeneration.

### Muse treatment increases Paneth cell proliferation associated with activated IL-6/Stat3 signaling pathway

**Fig. 6:**
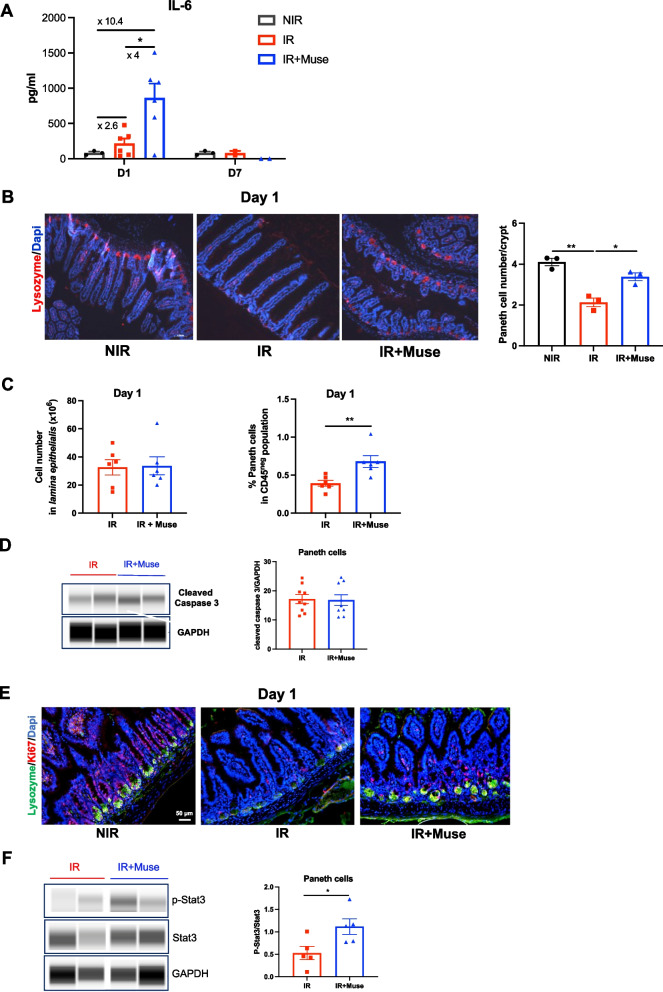
Muse enhance Paneth cell proliferation and IL-6/Stat3 signaling pathway. **A** Histogram plots showing IL-6 level in supernatant of 7-h-cultured intestine excised from non-treated or Muse-treated mice 1 or 7 days after irradiation, compared to non-irradiated mice. Data are represented with means ± SEM, *p < 0.05 (two-tailed Mann–Whitney U test). **B** Representative immunofluorescence images showing Lysozyme-positive cells (red) in the ileum of non-treated or Muse-treated mice 1 day after irradiation, compared to non-irradiated mice. Nuclei were counterstained with DAPI (blue) (top). Histogram plots showing Paneth cell number per crypts (bottom). Data are represented with means ± SEM, *p < 0.05; **p < 0.01 (two-tailed Mann–Whitney U test). **C** Histograms plots showing total cell number in the *lamina epithelialis* fraction (left) and proportion of Paneth cells (CD24^hi^CD166^med/+^) among CD45^neg^ population (right) in non-treated or Muse-treated mice 1 day after irradiation. Data are represented with means ± SEM, **p < 0.01 (two-tailed Mann–Whitney U test). **D** Representative cropping western blot showing protein level of cleaved caspase-3 in Paneth cells isolated from two non-treated and two Muse-treated mice 1 day after irradiation (left). GAPDH was used as internal control. Full length blots are presented in Additional file 1: Fig. S4A. Quantitative analysis of cleaved caspase 3 protein level normalized to GAPDH (right). Data are represented with means ± SEM. **E** Representative immunofluorescence images showing Lysozyme-positive cells (green) and Ki67-positive cells (red) in ileum of non-treated or Muse-treated mice 1 day after irradiation, compared to non-irradiated mice. Nuclei were counterstained with DAPI (blue). **F** Representative cropping western blot showing protein levels of p-Stat3 and Stat3 in Paneth cells isolated from two non-treated and two Muse-treated mice 1 day after irradiation. GAPDH was used as internal control (left). Full length blots are presented in Additional file 1: Fig. S4B. Quantitative analysis of the p-Stat3/Stat3 ratio after normalization to GAPDH (right). Data are represented with means ± SEM. *p < 0.05 (two-tailed Mann–Whitney U test)

IL-6, another mediator of ISC microenvironment known for the survival of intestinal epithelial cells [46] and the regulation of Paneth cell number [35, 36] has been analyzed after Muse injection in GIS model. One day after the 18 Gy-abdominal IR, murine IL-6 protein level was increased 2.6-fold in untreated mice and was fourfold enhanced in Muse-treated mice compared to untreated ones (Fig. 6A). These increased expressions of IL-6 were transient as IL-6 protein levels returned to normal 7 days after IR (Fig. 6A). The higher level of IL-6 prompted us to investigate the capacity of Muse to regulate the pool of Paneth cells. A twofold decrease of the number of lysozyme-positive Paneth cells per crypt was found in untreated mice 1 day after IR but not in Muse-treated mice indicating a better maintenance of Paneth cells per crypt (Fig. 6B).

To further analyze how Muse act on Paneth cells, we isolated by flow cytometry a Paneth cell- enriched fraction from *lamina epithelialis* using gating strategy described in Additional file 1: Fig. S3A [37] and characterized by a high expression of Lysozyme (Additional file 1: Fig. S3A–B). One day after IR, whereas the *lamina epithelialis* total cell number was not different (Fig. 6C, left panel), the frequency of Paneth cells was 1.7-fold higher in Muse-treated mice than in untreated mice (Fig. 6C, right panel) indicating that Muse can inhibit the radio-induced apoptosis of Paneth cells and/or increase Paneth cell proliferation. One day post-IR, WES experiment showed a similar expression level of cleaved caspase 3 protein between untreated and Muse-treated mice, indicated no effect of Muse on Paneth cell apoptosis (Fig. 6D and Additional file 1: Fig. S4A). In contrast, Paneth cell proliferation was increased in Muse-treated mice (Fig. 6E). As IL-6 regulates proliferation of Paneth cells through Stat3 activation [46], Stat3 and phospho-Stat3 (p-Stat3) protein levels were quantified by WES in a sorted population enriched in Paneth cells 1 day post-IR from untreated or Muse-treated mice. A twofold increase of the p-Stat3/Stat3 ratio was found in Muse-treated mice (Fig. 6F and Additional file 1: Fig. S4B).

Altogether, these results demonstrated that in this GIS model Muse could activate the IL-6/Stat3 signaling pathway in Paneth cells and increased their proliferation.

## Discussion

The intestine is one of the most radiosensitive organs in the body, and high doses of IR after accidental or therapeutic exposure lead to a GIS with severe intestinal damages, including loss of epithelial stem cells, and a high mortality rate [39]. There is currently no effective treatment that prevents and/or reduces GIS.

MSC-based therapy have been previously shown as a pre-clinical approach to stimulate epithelial stem cells to repair radio-induced intestinal injury [47]. Indeed, previous studies have shown that injection of MSC into irradiated mice resulted in an improved regeneration of intestinal or colonic tissues increasing the animal survival. Their effectiveness is essentially based on their secretion of growth factors with anti-inflammatory and pro-angiogenic effects but requires 1–5 million injected MSC per mouse within 2–24 h after IR [48]. Thus, in vitro amplification of MSC by weeks of culture limits their use for immediate treatment of emergency conditions. In MSC clinical trials, the dosages of MSC are typically described in cells/kg body weight (0.5–12 × 10^6^ cells/kg through single or multiple doses) [49]. Extensive expansion by in vitro cultures, cryo-preservation and thawing, and long-term storage of MSC may represent serious constraints and cost. Moreover, these processes may decrease the therapeutic efficacy of MSC [50, 51]. Thus, several priming approaches or genetically modified MSC have been proposed to improve the migration, homing, survival and function of MSC [52]. More recently, Bensemmane et al. showed that injection of stromal vascular fraction (SVF) from adipose tissue which takes only 4 h for preparation mitigates the GIS [35]. Nevertheless, this fraction which contains heterogeneous cell populations requires the injection of 2 million cells per mouse.

The discovery of Muse have stimulated renewed interest in stem cell-based therapy application due to their higher efficient therapeutic potential with a lower cell administration than MSC (2.1 × 10^5^ cells/kg) [15]. Clinical trials have been performed by intravenous injection of donor-derived Muse without HLA-matching and immunosuppressive treatment [15]. Currently, no data describe Muse-based therapy for radiation-induced pathologies and in particular in radio-induced GIS. In our study, we showed that only 50,000 WJ-Muse are sufficient to obtain the same therapeutic benefit on GIS, making a major advantage of Muse over MSC/SVF cells. Moreover, as Muse treatment is efficient only when cells are injected short time (< 24 h) after IR, a rapid management of patient could occur thanks to an allogeneic engraftment with cryo-preserved cells, that we showed to have the same therapeutic capacity than freshly isolated Muse.

Muse can be directly isolated from human bone marrow [36] and human connective tissues as skin [37], adipose tissue [38] and more recently from umbilical cord [39]. They are also collectable from MSC after amplification in culture. To date, the best known and the most commonly used sources of Muse are the adult bone marrow and the adipose tissue [40]. However, Muse collection from these two tissues requires invasive procedures. In contrast, the collect and isolation of birth-associated tissues including umbilical cord is easy and safe for both mother and child, and these fetal tissues are presently approved as a therapeutic source of stem cells [41]. Nevertheless, the therapeutic potential of Muse purified from umbilical cord matrix Wharton's jelly (WJ) is not currently documented. Here, we show that a large amount of Muse can be obtained after amplification of WJ-MSC, indicating a potential large-scale production of Muse for clinical trials. WJ- and BM-Muse shared the similar basic characteristics including expression of mesenchymal and pluripotent markers and differentiation potential. However, WJ-Muse have interesting specific properties suitable for the integration and homing of cells and therefore suitable for the intestinal regenerative process during GIS. Indeed, WJ-Muse expressed a higher level of the pluripotent markers Nanog, Oct3/4 and Sox2, characteristic of a more primitive status that limits a transplantation rejection [53]. They exhibit immunosuppressive properties and they expressed proteins implicated in intestinal barrier function such as ICAM-1 protein. Injection of MSC overexpressing ICAM-1 in mice with an inflammatory bowel disease (IBD) reduced inflammatory damage by promoting their homing to the colon [54]. Sumagin et al. found that in IBD disease, the interaction between epithelial cells expressing ICAM-1 and migrating polymorphonuclear leukocytes (PMN) resulted in epithelial cell proliferation and intestinal wound healing [29]. All these characteristics contribute to the migration and homing of Muse to repair the injured tissue.

In our model, Muse migrate into irradiated small intestine and persist into the crypts. They promote the regeneration of intestinal epithelium characterized by (1) a hyperproliferation of crypt cells, (2) an increased expression of the tight junction protein ZO-1 and (3) an increased expression of adherent protein EpCAM. The epithelium regeneration after injury is dependent on ISC survival and on the response of surrounding microenvironment that constitutes the ISC niche. This niche is composed of multiple cells as Paneth cells and immune cells including macrophages, providing growth factors, cytokines, and ligands that modulate the survival, differentiation or proliferation of ISC in homeostatic condition and after injury. In our study, we show that WJ-Muse have a major role during the acute phase of response to IR, as Muse rapidly enhance the secretion of murine MCP-1 and IL-6 cytokines, promoting a protective immune response and a pro-regenerative microenvironment. After Muse injection, we show that the enhancement of murine MCP-1 secretion is associated with an increase in the recruitment of monocytes into the inflamed small intestine. Moreover, we observed a M2-like macrophage invasion of intestinal tissue 7 days post-IR, which could limit intestinal inflammatory damage and promote repair and tissue regeneration. Several studies support a key role of macrophages in crypt regeneration. Transplantation of bone marrow-derived adherent stromal cells depleted of all myeloid cells failed to reconstitute the irradiated ISC niche and ISC regeneration leading to death of GIS mice. Depletion of host macrophages with clodronate resulted in poor survival after IR [44, 45]. In vitro, we demonstrated that Muse have the competence to modify the immunological reactions by limiting T-cell proliferation or by inducing polarization of macrophage into an anti-inflammatory M2-like phenotype. In fact, in vivo, Muse could challenge the inflammatory microenvironment generated after IR through, either their constitutive expression of factors such as HLA-G5, or their expression of factors such as IDO, Cox2, PD-L1 and TGFβ1 whose expression is under direct influence of the recipient’s inflammatory status. HLA-G5 and PD-L1 are known to reduce inflammation and immune responses and to display tolerogenic properties through interactions with inhibitory receptors on immune cells [55, 56]. IDO mediates the differentiation of monocytes into immunosuppressive M2 macrophages, which in turn contribute to T-cell suppression [48]. Prostaglandins synthesized from arachidonic acid by Cox2 suppressed radio-induced crypt apoptosis and enhanced crypt regeneration [49].

Our study showed likewise that Muse injection mediates a rapid increased level of murine IL-6. In the small intestine, IL-6 has been shown to protect intestinal epithelial cells from apoptosis during prolonged inflammation [57, 58]. Kuhn et al. showed the beneficial early IL-6 secretion in part produced by intra-epithelial lymphocytes for epithelial proliferation and intestinal wound healing after acute inflammatory injury [59]. Murine IL-6 production in Muse-treated mice during the acute inflammatory response is associated with an elevation of Paneth cell number 1 day post-IR, due to their highly enhanced proliferation through Stat3 signaling pathway activation. These findings are in agreement with previous data demonstrating that IL-6 is important for intestinal epithelial cell survival during acute inflammatory response after focal IR [60]. The IL-6/Stat3 signaling pathway regulates the Paneth cell number/proliferation through IL-6 receptor located on their basal membrane thereby increasing the proliferation of ISC [46]. Several studies showed that after radio-induced injury, Paneth cells participate actively to the intestinal tissue repair through their dedifferentiation into Lgr5^+^ ISC [61, 62] or facilitate ISC recovery by providing several niche factors such as wnt3a and metabolic intermediates [63–65].

## Conclusions

During the last 10 years, many preclinical studies have revealed the potential of Muse as a therapeutic effect in tissue repair and regenerative medicine. However, there are no data showing the involvement of Muse in regeneration of intestinal tissue after IR. This is the first demonstration of a therapeutic effect of fetal Muse in a preclinical model of radio-induced pathology. Our results indicate that Muse injection may be a feasible strategy to rapidly manage GIS due to their high regenerative capacity with only few cells injected.

### Supplementary Information

**Additional file: Fig. S1. A** Representative flow cytometry plots showing the viability (Hoechst) and phenotype (CD45, CD44, CD105, CD90 and CD73) of WJ-MSC before culture amplification. **B** Representative flow cytometry plots showing the percentage of SSEA-3^+^ Muse cells obtained after amplification of BM-MSC or WJ-MSC at passage 7. **C** Illustration of BM-Muse and WJ-Muse after culture in adherence condition (top) and BM-Muse and WJ-Muse clusters spontaneously obtained after culture in methylcellulose (bottom). **D** Volcano plot displaying the differential abundance of proteins in BM-Muse and WJ-Muse cells analyzed by MS-based label-free quantitative proteomics. Green and blue dots represent proteins found significantly enriched, respectively, in BM-Muse and WJ-Muse cells (fold change ≥ 2 and *p* value ≤ 0.005, leading to a Benjamini–Hochberg FDR < 1%). **E** Comparative analysis of common biological processes occurring in BM-Muse and WJ-Muse cells, identified by proteomic analysis. **Fig. S2 (relative to **Fig. 3**). A** Representative flow cytometry plots showing the viability (Hoechst) of thawed WJ-Muse cells and their SSEA-3 marker expression maintenance. **B** Kaplan–Meier survival analysis (upper panels) and weight loss changes (lower panels) for 30 days of 18 Gy abdominal exposed mice receiving either 50,000 freshly isolated Muse cells (left panels) or 50,000 cryo-preserved Muse cells (right panels) 4 h after irradiation. Statistical difference in survival between groups was determined by Log-rank (Mantel-Cox) test; *p ≤ 0.05; **p ≤ 0.01. Weight data are represented with means ± SEM. **Fig. S3 (relative to **Fig. 6**). A** Representative flow cytometry gating strategy for analyze and isolation of *lamina epithelialis* subpopulations enriched in stem cells (green box) or Paneth cells (red box). **B** Quantitative RT-qPCR analysis showing the expression markers of stem cells (Lgr5, Olfm4, Ascl2) and Paneth cells (Lysozyme) in isolated subpopulations, compared to CD24/CD166 double negative intestinal epithelial cells (black box). **Fig. S4 (relative to **Fig. 6**). A** Representative full-length WES showing the protein level of cleaved caspase-3 in Paneth cells isolated from non-treated and Muse-treated mice at 1 day after irradiation. GAPDH was used as an internal control. The framed areas correspond to the illustration of the cropping gel in the Fig. 6D. **B** Representative full-length WES showing the protein levels of p-Stat3 and Stat3 in Paneth cells isolated from non-treated and Muse-treated mice at 1 day after irradiation. GAPDH was used as an internal control. LPS-treated RAW cells were used as p-Stat3 positive control (CTL). The framed areas correspond to the illustration of the cropping gel in the Fig. 6F.

## Data Availability

The mass spectrometry proteomics data have been deposited to the ProteomeXchange Consortium via the PRIDE partner repository with the dataset identifier PXD036883. All relevant data and material to reproduce the findings are available in the manuscript and will be available upon request.

## Abbreviation

Muse: Multilineage-differentiating stress enduring
MSC: Mesenchymal stem cells
Gy: Gray
GIS: Gastrointestinal syndrome
IR: Irradiation
WJ: Wharton’s jelly
BM: Bone marrow
LE: Lamina epithelialis
LP: Lamina propria
ISC: Intestinal stem cells
SSEA-3: Stage-specific embryonic antigen 3
BMDM: Bone marrow-derived macrophages
IEC: Intestinal epithelium cells
hPBMC: Human peripheral blood mononuclear cells
mSL: Mouse spleen lymphocytes
