# Supplementary material for: Fetal Muse-based therapy prevents lethal radio-induced gastrointestinal syndrome by intestinal regeneration
Source: Stem Cell Res Ther. 2023 Aug 11;14:201. doi: 10.1186/s13287-023-03425-1 (PMC10416451; doi:10.1186/s13287-023-03425-1)

Supplemental Figure 1

A

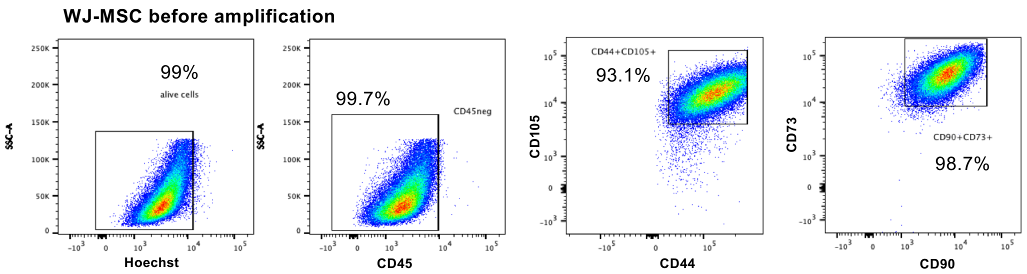

B

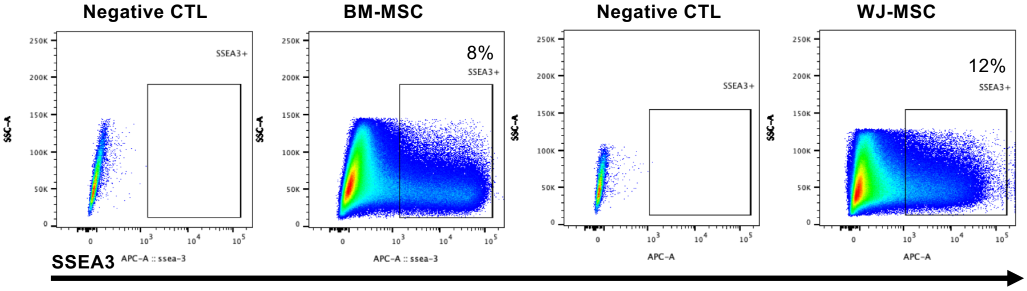

C

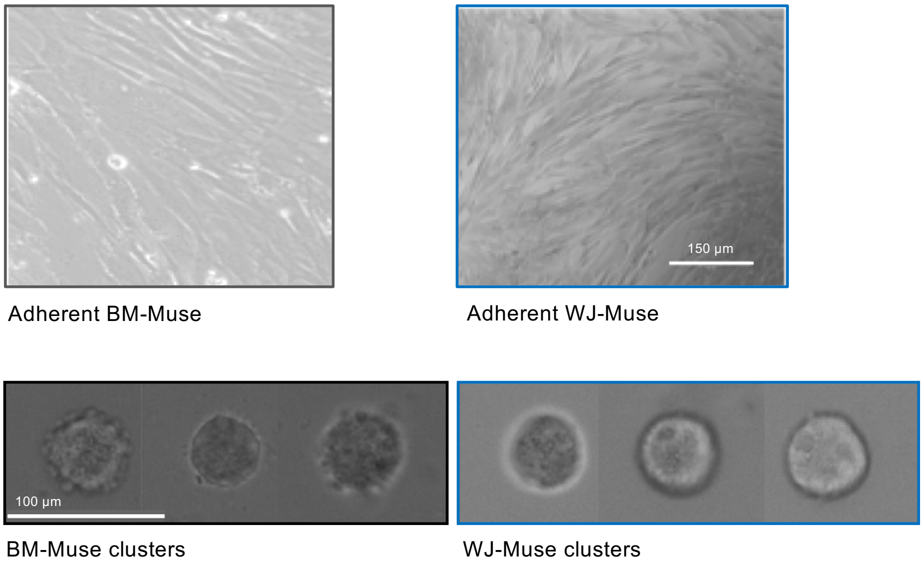

D

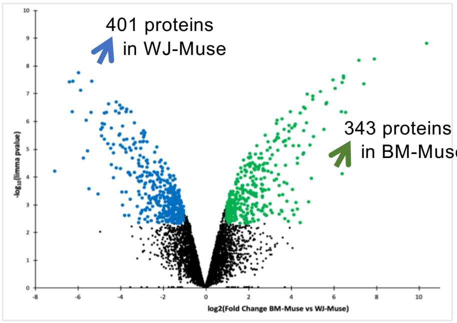

E

BM-Muse

GO term	BIOLOGICAL Process	Nb proteins	P-Value	Fold enrichment
GO:0001525	ANGIOGENESIS	12	1.10E-03	1.2E+01
GO:0007155	CELL ADHESION	20	5.35E-04	2.50E+00
GO:0016477	CELL MIGRATION	11	5.64E-03	3.70E+00
GO:0042493	RESPONSE TO DRUG	14	4.13E-04	3.20E+00

WJ-Muse

GO term	BIOLOGICAL Process	Nb proteins	P-Value	Fold enrichment
GO:0001525	ANGIOGENESIS	12	6.30E-03	2.63E+00
GO:0007155	CELL ADHESION	19	1.42E-02	1.86E+00
GO:0030335	POSITIVE REGULATION OF CELL MIGRATION	10	4.84E-02	2.11E+00
GO:0042493	RESPONSE TO DRUG	11	4.60E-02	2.03E+00

Supplemental Figure 2 (relative to Figure 3)

A

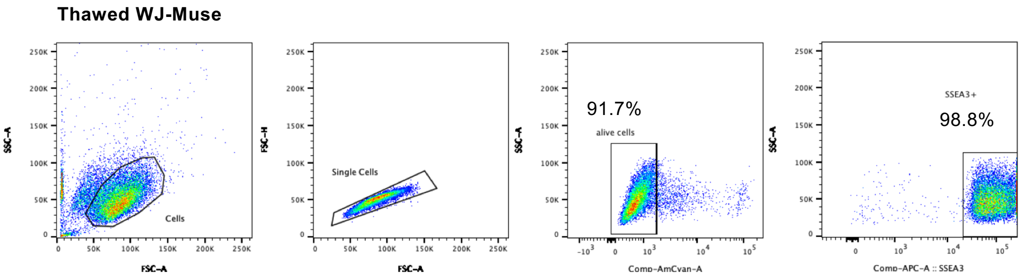

B

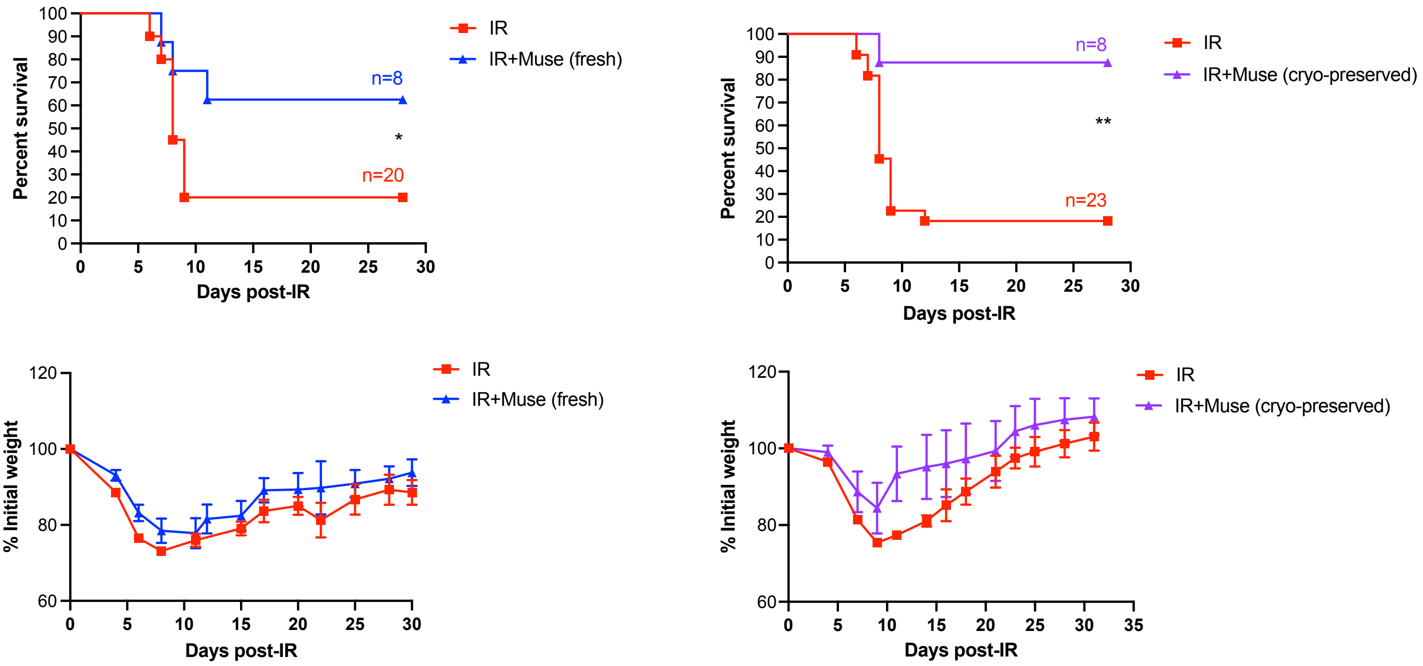

Supplemental Figure 3 (Relative to Figure 6)

A

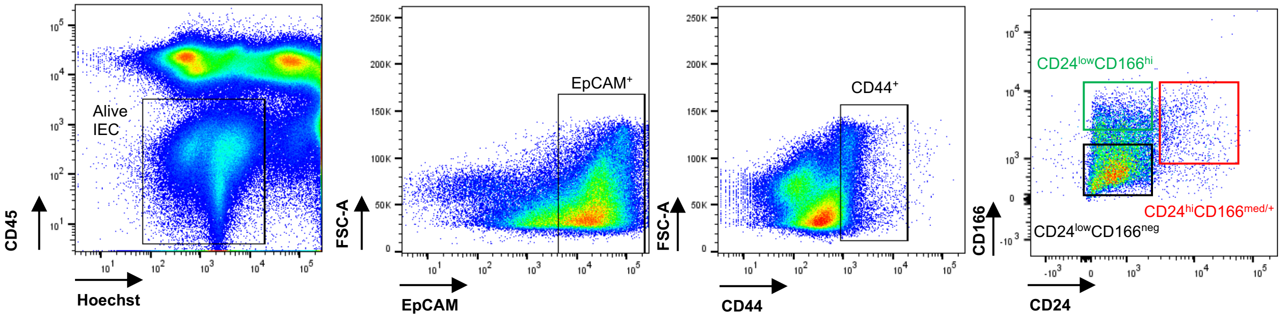

B

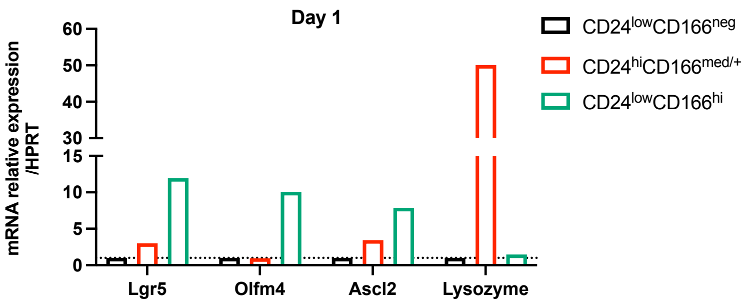

Supplemental Figure 4 (Relative to Figure 6)

A

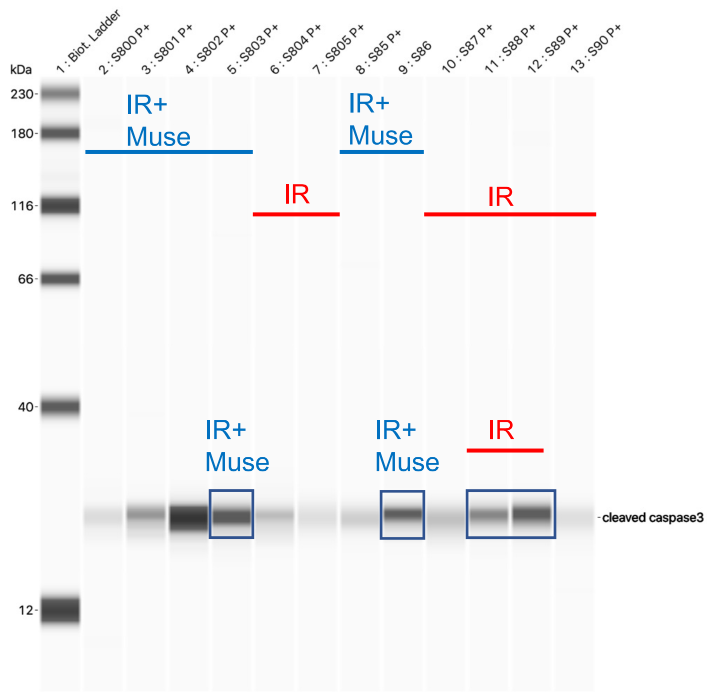

B

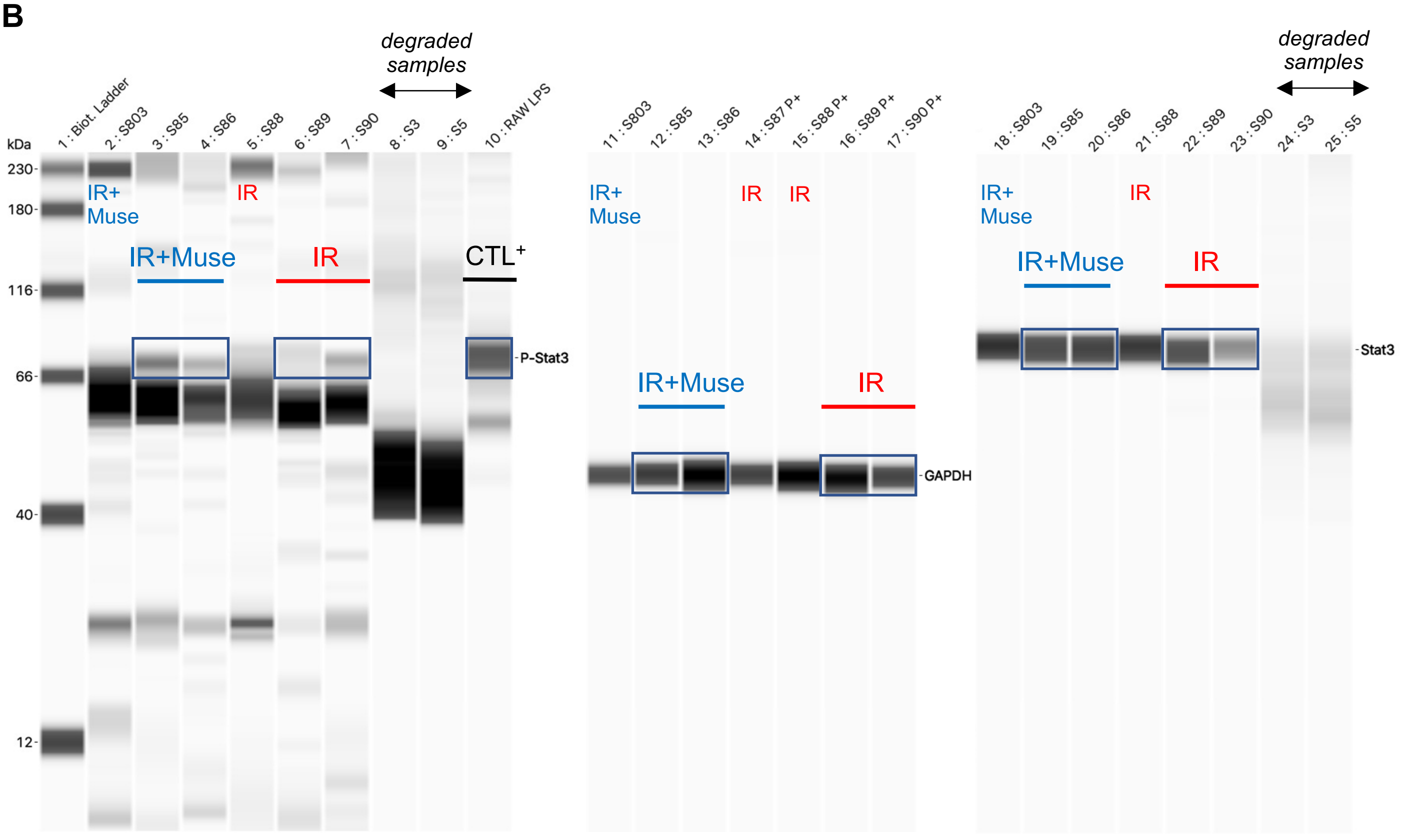

Supplement: Supplementary file 1 — Additional file: Fig. S1. A Representative flow cytometry plots showing the viability (Hoechst) and phenotype (CD45, CD44, CD105, CD90 and CD73) of WJ-MSC before culture amplification. B Representative flow cytometry plots showing the percentage of SSEA-3+ Muse cells obtained after amplification of BM-MSC or WJ-MSC at passage 7. C Illustration of BM-Muse and WJ-Muse after culture in adherence condition (top) and BM-Muse and WJ-Muse clusters spontaneously obtained after culture in methylcellulose (bottom). D Volcano plot displaying the differential abundance of proteins in BM-Muse and WJ-Muse cells analyzed by MS-based label-free quantitative proteomics. Green and blue dots represent proteins found significantly enriched, respectively, in BM-Muse and WJ-Muse cells (fold change ≥ 2 and p value ≤ 0.005, leading to a Benjamini–Hochberg FDR < 1%). E Comparative analysis of common biological processes occurring in BM-Muse and WJ-Muse cells, identified by proteomic analysis. Fig. S2 (relative to Fig. 3). A Representative flow cytometry plots showing the viability (Hoechst) of thawed WJ-Muse cells and their SSEA-3 marker expression maintenance. B Kaplan–Meier survival analysis (upper panels) and weight loss changes (lower panels) for 30 days of 18 Gy abdominal exposed mice receiving either 50,000 freshly isolated Muse cells (left panels) or 50,000 cryo-preserved Muse cells (right panels) 4 h after irradiation. Statistical difference in survival between groups was determined by Log-rank (Mantel-Cox) test; *p ≤ 0.05; **p ≤ 0.01. Weight data are represented with means ± SEM. Fig. S3 (relative to Fig. 6). A Representative flow cytometry gating strategy for analyze and isolation of lamina epithelialis subpopulations enriched in stem cells (green box) or Paneth cells (red box). B Quantitative RT-qPCR analysis showing the expression markers of stem cells (Lgr5, Olfm4, Ascl2) and Paneth cells (Lysozyme) in isolated subpopulations, compared to CD24/CD166 double negative intestinal e [file 13287_2023_3425_MOESM1_ESM.pdf]
